# The Efficacy and Safety of Laparoscopy for Blunt Abdominal Trauma: A Systematic Review and Meta-Analysis

**DOI:** 10.3390/jcm10091853

**Published:** 2021-04-24

**Authors:** Young-Jun Ki, Young-Goun Jo, Yun-Chul Park, Wu-Seong Kang

**Affiliations:** 1 Division of Acute Care Surgery, Department of Surgery, Asan Medical Center, University of Ulsan College of Medicine, Seoul 05505, Korea; kyj8302@hanmail.net; 2 Division of Trauma, Department of Surgery, Chonnam National University Medical School and Hospital, Chonnam National University, Gwangju 61469, Korea; thinkjo82@gmail.com (Y.-G.J.); kontikithor@gmail.com (Y.-C.P.); 3 Department of Trauma Surgery, Jeju Regional Trauma Center, Cheju Halla General Hospital, Jeju 63127, Korea

**Keywords:** laparoscopy, laparotomy, blunt trauma, penetrating trauma, meta-analysis

## Abstract

The efficacy and safety of laparoscopy for blunt trauma remain controversial. This systemic review and meta-analysis aimed to evaluate the usefulness of laparoscopy in blunt trauma. The PubMed, EMBASE, and Cochrane databases were searched up to 23 February 2021. Meta-analyses were performed using odds ratios (ORs), standardized mean differences (SMDs), and overall proportions. Overall, 19 studies with a total of 1520 patients were included. All patients were hemodynamically stable. In the laparoscopy group, meta-analysis showed lesser blood loss (SMD −0.28, 95% confidence interval (CI) −0.51 to −0.05, I^2^ = 62%) and shorter hospital stay (SMD −0.67, 95% CI −0.90 to −0.43, I^2^ = 47%) compared with the laparotomy group. Pooled prevalence of missed injury (0.003 (95% CI 0 to 0.023), I^2^ = 0%), nontherapeutic laparotomy (0.004 (95% CI 0.001 to 0.026), I^2^ = 0%), and mortality (0.021 (95% CI 0.010 to 0.043), I^2^ = 0%) were very low in blunt trauma. In subgroup analysis, recently published studies (2011–present) showed lesser conversion rate (0.115 (95% CI 0.067 to 0.190) vs. 0.391 (95% CI 0.247 to 0.556), test for subgroup difference: *p* < 0.01). This meta-analysis suggests that laparoscopy is a safe and feasible option in hemodynamic stable patients with blunt abdominal trauma.

## 1. Introduction

The use of laparoscopy on patients with trauma was first reported in the 1970s [1]. In the initial stage, the purpose of laparoscopy was only the diagnosis, and consequent open laparotomy was performed in cases that needed further procedures such as vessel ligation, bowel resection, or suture. The early reports of laparoscopy focused on only the diagnostic role. Several studies have reported that diagnostic laparoscopy in patients with trauma has a high diagnostic accuracy of nearly 100% [2]. Diagnostic laparoscopy is beneficial in avoiding unnecessary nontherapeutic laparotomy, which is usually accompanied by more complications than laparoscopy [3]. Moreover, with the development of the laparoscopic technique and equipment, therapeutic laparoscopy has been attempted on patients with trauma. There has recently been an increase in the number of reports on therapeutic laparoscopy in patients with trauma [4]. Recently, the laparoscopic procedure comprises both diagnostic and therapeutic purposes. As the therapeutic role of laparoscopy is increasing, the indication is widening, and unnecessary laparotomy is decreasing.

In terms of injury mechanism, compared to penetrating abdominal trauma (PAT), few studies have been conducted on laparoscopy in patients with blunt abdominal trauma (BAT). This may be because there are many ambiguous blunt regions, such as hematoma and bruised organs. In addition, laparoscopy for BAT and PAT is mostly performed in hemodynamically stable patients. There is still controversy regarding the indications and safety of laparoscopy for BAT. There has been a substantial concern that it can be dangerous for patients with hemodynamic instability, and it can miss serious blunt injury despite its many advantages. Therefore, we conducted a systematic review and meta-analysis to evaluate the efficacy and safety of laparoscopy in patients with BAT.

## 2. Materials and Methods

### 2.1. Published Study Search and Selection Criteria

This study was conducted following the Preferred Reporting Items for Systematic Reviews and Meta-Analysis [5]. Relevant articles were obtained by searching the MEDLINE PubMed, EMBASE, and Cochrane databases up to 23 February 2021. These databases were searched using the following keywords: “(laparoscopy OR laparoscopic) AND (trauma OR traumatic).” In addition, we manually searched the reference lists of relevant articles. The titles and abstracts of all searched articles were screened for exclusion. Review articles and previous meta-analyses were also screened to identify additional eligible studies. The search results were then reviewed, and articles were included if the study investigated therapeutic laparoscopy for patients with blunt trauma.

The inclusion criteria for this review were as follows: (i) patients with blunt trauma, (ii) patients who underwent therapeutic laparoscopic surgery, (iii) comparison between laparoscopy and laparotomy or between blunt trauma and penetrating trauma, (iv) report of relevant outcomes such as operative and postoperative measurements, and (v) report of odds ratio (OR) or mean with standard deviation or provision of data for their calculation. Articles of studies on other diseases, non-original articles, articles that studied pediatric patients, or non-English language publications were excluded. Laparoscopy performed in an emergency department or intensive care unit without general anesthesia was excluded.

### 2.2. Data Extraction

Data from all eligible studies were extracted by two investigators. Data extracted from each eligible study include the following [6,7,8,9,10,11,12,13,14,15,16,17,18,19,20,21,22,23,24]: name of the first author, year of publication, study location, study design, study period, number of patients analyzed, age of patients, injury severity score (ISS), operation time, volume of intraoperative blood loss, rate of conversion to open laparotomy, missed injury, nontherapeutic laparotomy, duration of hospital stay, overall complications, rate of wound infection, and mortality rate. Conversion to open laparotomy was defined as laparotomy after initial laparoscopy during the same operation. Nontherapeutic laparotomy was defined as no additional therapeutic procedure during laparotomy because of minimal or no injured organ.

### 2.3. Quality Assessment

The Newcastle-Ottawa quality assessment scale (NOS) was used to evaluate the risk of bias in the observational studies [25]. The NOS uses a star system with the following three domains: selection, comparability, and exposure/outcome. All studies were independently reviewed by two investigators. Any disagreement concerning study selection and data extraction was resolved through consensus.

### 2.4. Statistical Analysis

All statistical analyses were performed using the “meta” package of the R programming language, version 4.0.3 (R foundation, Vienna, Austria). The meta-analyses were performed using ORs for binary outcomes and standardized mean differences (SMDs) for continuous outcome measures and overall proportion for single proportional outcomes. Pooled analysis was performed using the inverse variance method with random effects weighing for meta-analysis of outcomes. Heterogeneity was assessed through visual inspection of the forest plots and estimated by using I^2^ statistics and Cochran’s Q (Chi-square test) (results with *p*-value < 0.10 were considered significant). I^2^ statistics of >25%, >50%, and >75% were considered to represent low, moderate, and high heterogeneity, respectively [26]. Due to the few eligible studies (<20), we could not assess publication bias using statistical methods (e.g., funnel plots and Egger regression test) [27].

We performed subgroup analysis to assess the between-study heterogeneity. We divided the study groups by two moderators, such as the year of publication and type of injured organ. In terms of publication year, we defined two groups as follows: before 2010 vs. 2011 to present. The type of injured organ was categorized as follows: “general abdominal organ” referred to all general abdominal organs, including solid and hollow viscus organ. “Solid organ” referred to the liver or spleen. “Hollow viscus organ” referred to the upper or lower gastrointestinal tract. We did not conduct the subgroup analysis unless there was sufficient statistical power (small number of studies, k < 10).

We performed sensitivity analysis after excluding studies that comprised only the liver or spleen because they included substantially heterogenous indications. If the results did not change significantly after excluding those studies, then we considered that the results were robust. If the results changed significantly, we considered that the results were unstable.

## 3. Results

### 3.1. Selection and Characteristics

**Figure 1:**
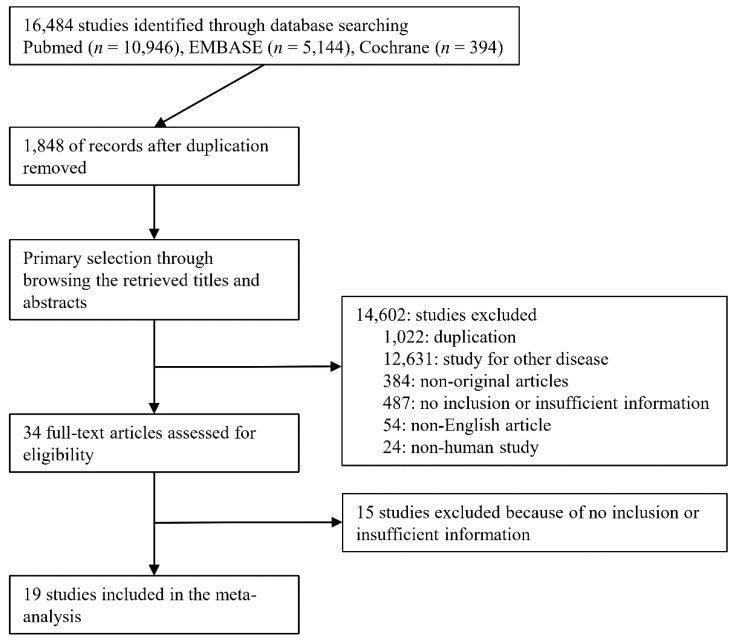
Flow diagram for identification of eligible studies.

Overall, 16,484 studies were identified through a search of the databases. Of these, 14,602 were excluded after the title and abstract review. Studies were excluded for the following reasons: duplicates (*n* = 1022), non-original studies (*n* = 384), studies on other diseases (*n* = 12,631), non-human studies (*n* = 24), and studies in non-English languages (*n* = 54). Finally, 19 studies with a total of 1520 patients were included in this meta-analysis after full-text review (Figure 1). Detailed information on the eligible studies is shown in Table 1.

All 19 eligible studies were single-center retrospective observational studies (Table 1). Ten studies [7,9,10,11,12,15,18,19,20,23] comprised only BAT, and the remaining nine [6,8,13,14,16,17,21,22,24] included both BAT and PAT. All the studies were on abdominal injuries: four studies included hollow viscous organ injuries [9,10,11,23], two [12,19] included only spleen injuries, and one [21] included a non-descriptive injury. Five studies compared the laparoscopy and laparotomy group [10,13,17,19,20], whereas seven studies compared laparoscopy for BAT with laparoscopy for PAT [6,8,14,16,21,22,24]. Six studies that were not comparable comprised only single proportional outcomes [7,9,11,12,15,18].

### 3.2. Quality Assessment

All the included studies were observational studies. The quality assessment and risk of bias for each eligible study are summarized in Table 2. According to the NOS system, we found that all studies had an insufficient selection of controls in the selection domain and non-response rate in the exposure domain. Overall, most studies had relatively high scores on using NOS that varied from 5 to 7 points. However, potential confounding factors may exist regarding selection and exposure. In terms of study design and anatomic location of trauma, there was substantial heterogeneity across the studies (Table 1).

### 3.3. BAT and PAT

**Figure 2:**
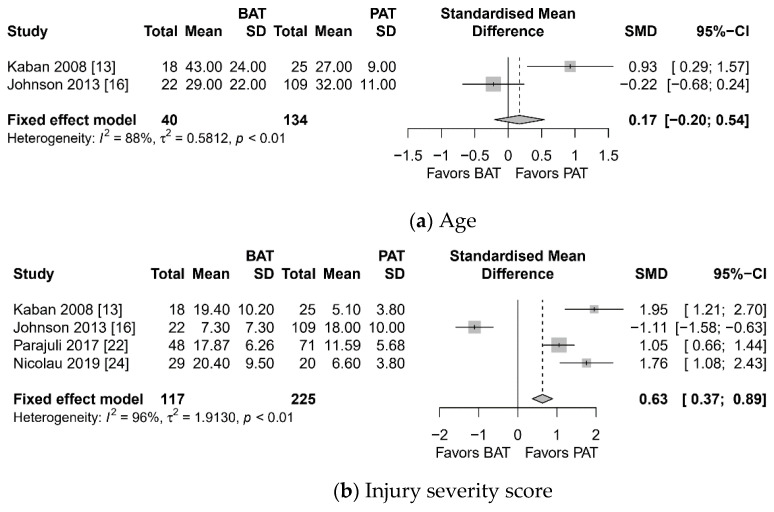

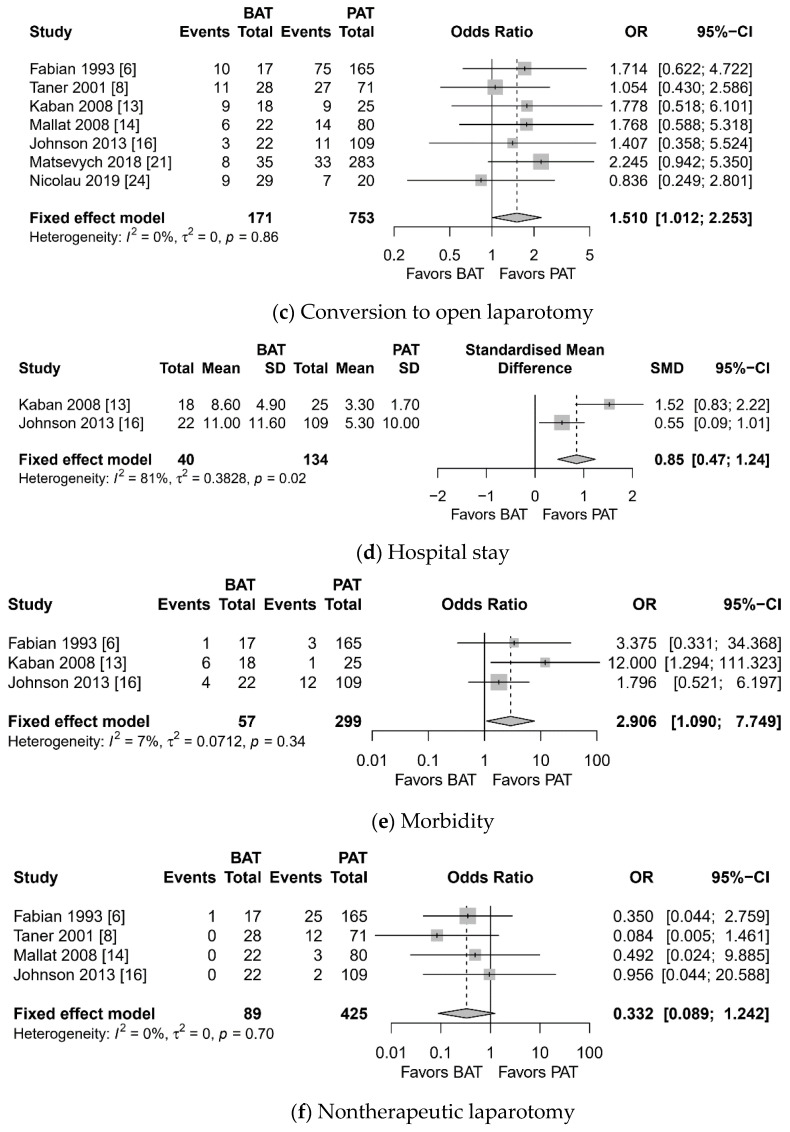
Forest plot: blunt abdominal trauma (BAT) vs. penetrating abdominal trauma (PAT). (**a**) Age, (**b**) injury severity score, (**c**) conversion to open laparotomy, (**d**) hospital stay, (**e**) morbidity, (**f**) nontherapeutic laparotomy.

Meta-analysis showed higher ISS (SMD 0.63, 95% confidence interval (CI) 0.37 to 0.89, I^2^ = 96%), more conversion to laparotomy (OR 1.510, 95% CI 1.012 to 2.253, I^2^ = 0%), longer hospital stay (SMD 0.85, 95% CI 0.47 to 1.24), and more morbidity (OR 2.906, 95% CI 1.090 to 7.749, I^2^ = 7%) in the BAT group than in PAT (Figure 2). However, there was no significant difference in terms of age and nontherapeutic laparotomy.

### 3.4. Laparoscopy and Laparotomy

**Figure 3:**
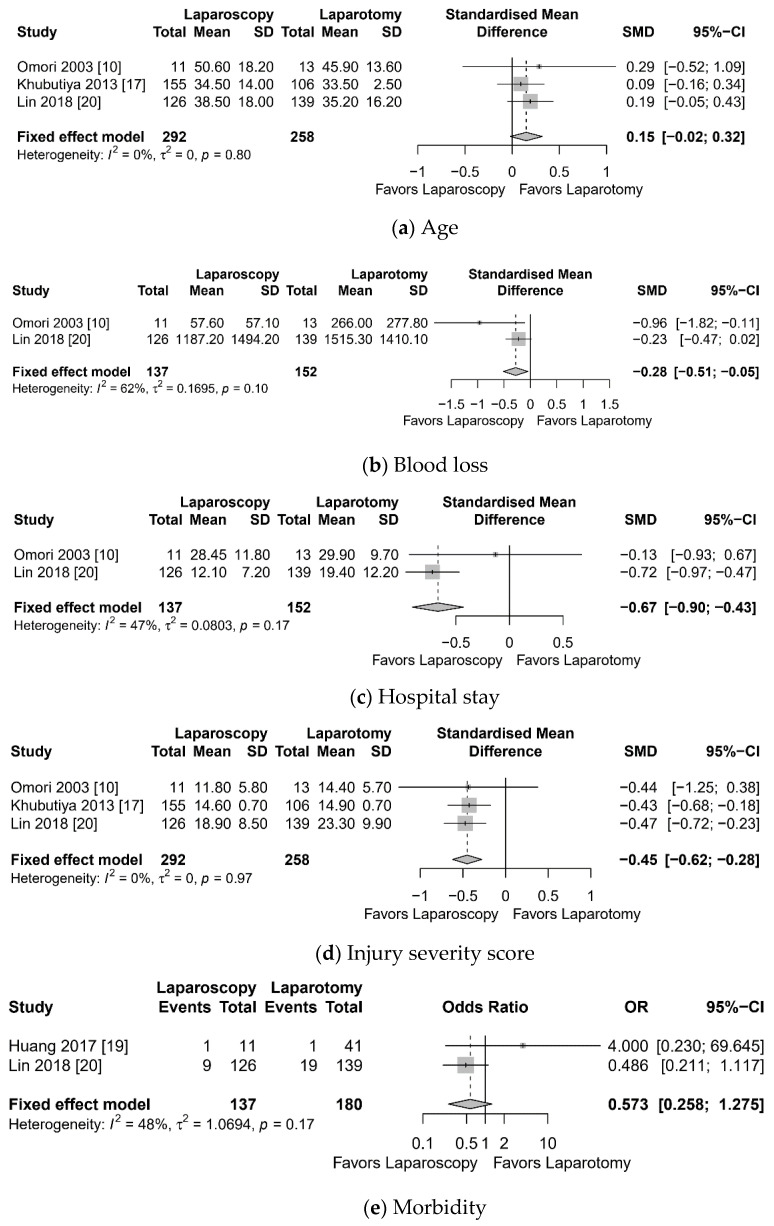

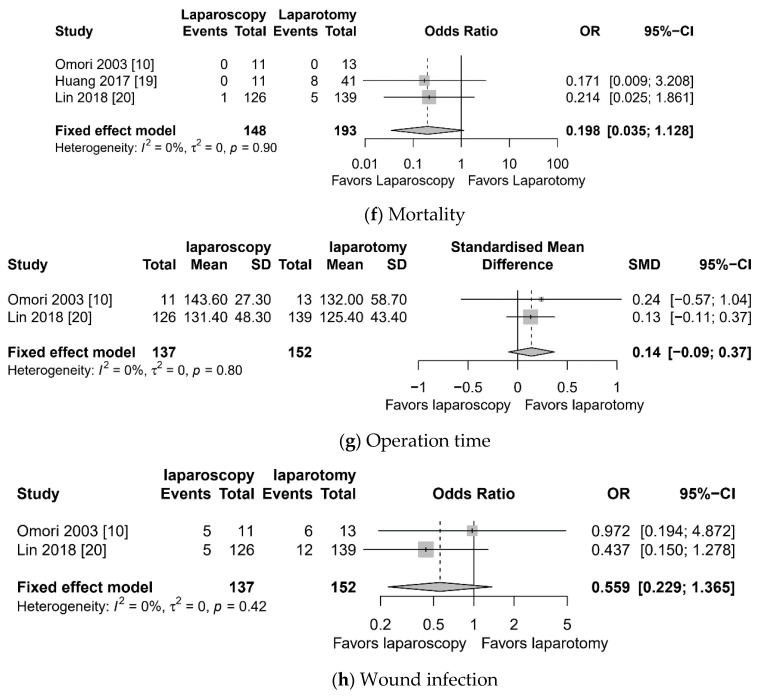
Forest plot: laparoscopy vs. laparotomy for blunt abdominal trauma. (**a**) age, (**b**) blood loss, (**c**) hospital stay, (**d**) injury severity score, (**e**) morbidity, (**f**) mortality, (**g**) operation time, (**h**) wound infection.

Meta-analysis showed lesser blood loss (SMD −0.28, 95% CI −0.51 to −0.05, I^2^ = 62%), shorter hospital stays (SMD −0.67, 95% CI −0.90 to −0.43, I^2^ = 47%), and lesser ISS (SMD −0.45, 95% CI −0.62 to −0.28, I^2^ = 0%) in the laparoscopy group compared to the laparotomy group (Figure 3). However, there was no significant difference in terms of age, morbidity, mortality, and operation time.

### 3.5. Prevalence of Conversion to Laparotomy, Missed Injury, Nontherapeutic Laparotomy, Morbidity, and Mortality

**Figure 4:**
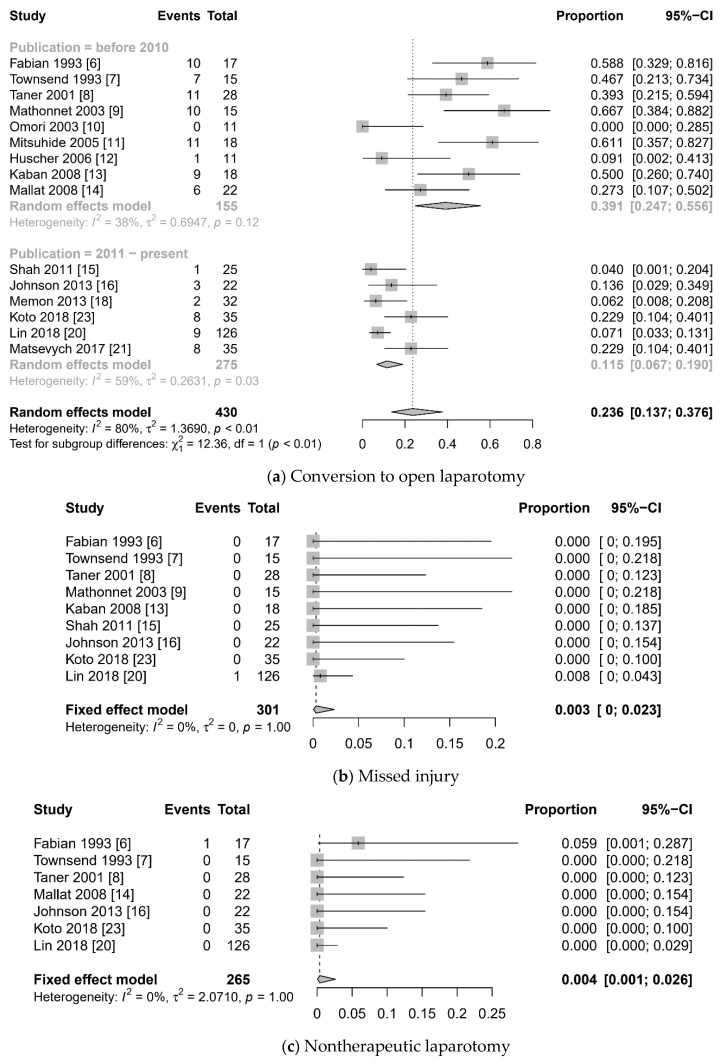

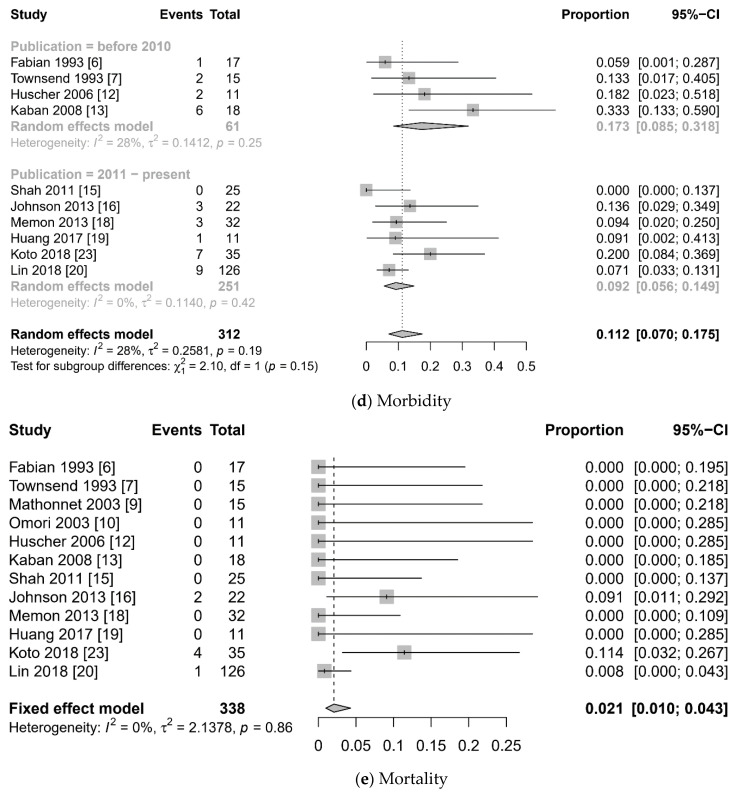
Forest plot: laparoscopy for blunt abdominal trauma. (**a**) conversion to open laparotomy, (**b**) missed injury, (**c**) nontherapeutic laparotomy, (**d**) morbidity, (**e**) mortality.

The meta-analysis of the prevalence of perioperative outcomes is summarized in Figure 4. Overall pooled prevalence of conversion to laparotomy was 0.236 (95% CI 0.137 to 0.376, I^2^ = 80%). Notably, recently published studies (since 2011) showed lesser conversion rate (0.115 (95% CI 0.067 to 0.190) vs. 0.391 (95% CI 0.247 to 0.556), test for subgroup difference: *p* < 0.001). Pooled prevalence of missed injury (0.003 (95% CI 0 to 0.023), I^2^ = 0%), nontherapeutic laparotomy (0.004 (95% CI 0.001 to 0.026), I^2^ = 0%), and mortality (0.021 (95% CI 0.010 to 0.043), I^2^ = 0%) were very low.

### 3.6. Subgroup Analysis

Subgroup analysis showed a significant difference in the conversion rate when the publication year of the study was used as a moderator (Table 3) (test for subgroup difference: *p* < 0.01). However, the type of injured organ was not a significant moderator. Subgroup analysis for morbidity and mortality showed that there was no significant difference in terms of publication year and injured organ.

### 3.7. Sensitivity Analysis

For sensitivity analysis, in terms of the prevalence of conversion rate, we deleted studies that comprised only solid organ injuries, and we obtained similar results as follows: pooled prevalence was 0.428 (95% CI 0.272 to 0.600) in the “before 2010” group and 0.115 (95% CI 0.067 to 0.190) in the “2011–present” group respectively, with a significant subgroup difference (*p* < 0.001). Using another moderator, injured organ, we obtained similar statistical results (general abdominal organ, pooled prevalence 0.202, 95% CI 0.105 to 0.353 vs. hollow viscus organ, pooled prevalence 0.3194, 95% CI 0.085 to 0.703). After deletion of those studies, we obtained similar statistical results in terms of missed injury (pooled prevalence 0.004, 95% CI 0.001 to 0.024, I^2^ = 0%), morbidity (pooled prevalence 0.107, 95% CI 0.058 to 0.189, I^2^ = 50%), mortality (pooled prevalence 0.023, 95% CI 0.011 to 0.048, I^2^ = 0%), and nontherapeutic laparotomy (pooled prevalence 0.004, 95% CI 0.001 to 0.028, I^2^ = 0%).

## 4. Discussion

Our meta-analysis suggests the favorable outcomes of laparoscopy for BAT. We found several clinically important features of laparoscopy. First, similar to the non-trauma field, our meta-analysis showed that laparoscopy had advantages in terms of blood loss during surgery and hospital stay compared to laparotomy. Second, laparoscopy in BAT compared to PAT had a higher morbidity and conversion rate. Third, the overall prevalence of missed injury, nontherapeutic laparotomy, morbidity, and mortality was very low and acceptable in BAT rather than PAT. Fourth, the conversion rate in recent studies improved more than that in previous studies. Finally, in the eligible studies included in our analysis, laparoscopy was limited to patients without hemodynamic instability or extensive trauma. Despite the substantial heterogeneity and risk of bias, our study has significant implication to trauma surgeons.

Several previous systematic reviews and meta-analyses have been conducted on laparoscopy for patients with trauma. In a systematic review and meta-analysis regarding laparoscopy in PAT [2] including 13 prospective and 38 retrospective studies, few therapeutic laparoscopies were included (13.8%). This review noted that laparoscopy had an important role in detecting and treating diaphragmatic injuries. Remarkably, the authors noted 83 missed injuries, indicating 66.7–100% sensitivity and 33–100% specificity. The eligible studies of the present meta-analysis reported only one missed injury. When operating on patients with trauma, it is crucial that injuries are not missed. The most recent systematic review and meta-analysis including 9817 laparotomies [4] demonstrated that the incidence of therapeutic laparotomy decreased from 69% to 47.5%, whereas the incidence of therapeutic laparoscopy increased from 7.2% to 22.7%. This review did not separate the outcomes of blunt trauma. In another meta-analysis that compared laparoscopy and laparotomy [28], it was reported that laparoscopy improved perioperative outcomes and reduced the risk of complications among hemodynamically stable patients with abdominal trauma. This analysis also did not separate the outcomes of blunt trauma and included many Chinese-written articles from a Chinese database. We did not include the Chinese database. To the best of our knowledge, our analysis is the first meta-analysis that analyzed the efficacy of laparoscopy for BAT. We also computed the pooled prevalence, and this significantly differed from previous studies [2,4,28].

Currently, laparoscopic surgery is widely accepted as a treatment for non-traumatic disease, and the controversy surrounding its technical issues has reduced. There have been considerable improvements in laparoscopic skill and laparoscopic equipment over the past few decades. This evolution was possible because of the development of various useful instruments, including high-resolution cameras, suturing devices, staplers, and energy devices, that allow for effective hemostasis and resection. In the subgroup analysis of our study, recent studies showed lesser conversion rate than early studies. In our study, several studies published at an early stage used laparoscopy for only diagnostic purposes [6,7,8]. However, recent studies are more in the realm of therapeutic laparoscopy, contributing to decreasing conversion rate. They included laparoscopic procedures such as bowel resection, bowel repair, bladder repair, splenectomy, distal pancreatectomy, diaphragm repair, and hemostasis [15,16,18,20,21,23]. In early studies, laparotomy was needed for these procedures. Large-scale randomized controlled trials have shown that, in terms of stomach and colon cancers, the outcomes of laparoscopic surgery are similar and non-inferior to the outcomes of open surgery [29,30,31]. This implies that little progress is required to solve the technical problems associated with bowel surgery. However, laparoscopic surgery on retroperitoneal organs, such as the duodenum and pancreas, remains controversial. No randomized controlled trials were reported in recent meta-analyses on pancreatic laparoscopic surgery [32,33]. In our analysis, no randomized controlled trials were found, and the bowel and mesentery were the most injured organs. The laparoscopic retroperitoneal approach requires greater experience and skill than the other approaches in bowel surgery. Thus, trauma surgeons need a great deal of experience and advanced skill. The possibility of open conversion may be high in severe bleeding and retroperitoneal organ injury [1]. Computed tomography (CT) or focused assessment with sonography for trauma (FAST) may help to decide to attempt therapeutic laparoscopy [1,34]. If there are no specific findings on physical examination and there is no severe bleeding in CT or FAST, therapeutic laparoscopy may be attempted. Recently, interventional radiology has evolved considerably for hemostasis in patients with hemorrhagic pelvic fracture, liver laceration, spleen rupture, or major vascular trauma [35,36,37,38].

Our analysis had several limitations. First, all the eligible studies were retrospective and observational; therefore, selection bias was inevitable. A prospective study is needed to determine the true effect size. Second, verification of publication bias was difficult because of the limited number of eligible studies. Third, we computed estimated prevalence by using single descriptive statistics because there were limited comparative studies. This may induce substantial heterogeneity. To overcome the weakness, we conducted subgroup analysis and sensitivity analysis. Fourth, only articles written in English were included. Fifth, we could not separate the effect size related to hemodynamic status even though hemodynamic stability is important for choosing the operative strategy. Finally, lower ISS in the laparoscopy group might be a confounder related to other effect sizes. The small number of studies including comparison of ISS is another limitation. However, lower ISS of the laparoscopy group in our study suggests that appropriate patient selection is crucial.

## 5. Conclusions

Laparoscopy for BAT showed favorable outcomes in terms of blood loss during surgery, hospital stay, missed injury, nontherapeutic laparotomy, and morbidity. The conversion rate has improved in recent studies. This meta-analysis suggests that laparoscopy is a safe and feasible option for BAT with hemodynamic stability. However, the retrospective nature and heterogeneity between studies make the generalization of the results of this meta-analysis limited. A large-scale multicenter prospective study is needed to determine the exact effect sizes of laparoscopy in BAT. However, such research design will be a big challenge in clinical practice.

## Figures and Tables

**Table 1:** Main characteristics of the eligible studies.

Study		Country	Study Design	Study Period	Type of Trauma	Anatomic Location of Trauma	Comparison	Number of Participants	Hemodynamic Status	Exclusion Criteria of Laparoscopy
Author	Year									
Fabian [6]	1993	United States	Observational, Single center	1990–1991	Blunt, penetrating	liver, spleen, stomach, small intestine, colon, mesentery, diaphragm, pancreas, duodenum, gall bladder, bladder, vascular	Blunt	17	Stable	Hemodynamic instability
							Stab, Gunshot	165		
Townsend [7]	1993	United States	Observational, Single center	1991–1992	Blunt	liver, spleen	None	15	Stable	Hemodynamic instability, peritonitis, head injury, <18 years, pregnancy, previous abdominal surgery
Taner [8]	2001	Turkey	Observational, Single center	1995–1999	Blunt, penetrating	General abdominal trauma	Blunt	28	Stable	Hemodynamic instability, peritonitis, head injury, <18 years, pregnancy, previous abdominal surgery
							Penetrating	71		
Mathonnet [9]	2003	France	Observational, Single center	1985–2001	Blunt	small intestine	None	15	Non-descriptive	Non-descriptive
Omori [10]	2003	Japan	Observational, Single center	1993–1997	Blunt	small intestine, colon	Laparoscopy	13	Stable	Hemodynamic instability
							Laparotomy	11	Stable	
Mitsuhide [11]	2005	Japan	Observational, Single center	1994–2002	Blunt	stomach, small intestine, colon	None	18	Stable	Hemodynamic instability, massive hemoperitomeum, injuries to abdominal organ other than bowel
Huscher [12]	2006	Italy	Observational, Single center	2000–2004	Blunt	Spleen	None	11	Stable	Non-descriptive
Kaban [13]	2008	United States	Observational, Single center	2001–2004	Blunt, penetrating	General abdominal trauma	laparoscopy	18	Stable	Non-descriptive
							laparotomy	25		
Mallat [14]	2008	United States	Observational, Single center	1996–2006	Blunt, penetrating	General abdominal trauma	Blunt	22	Stable	Non-descriptive
							Stab, Gunshot	80		
Shah [15]	2011	India	Observational, Single center	2004–2008	Blunt	liver, spleen, stomach, small intestine, colon, kidney	None	25	Stable	Hemodynamic instability, severe head injury, sever chest injury, compound fracture, spine fracture, anticipated difficult endotracheal intubation, pregnancy
Johnson [16]	2013	United States	Observational, Single center	2001–2010	Blunt, penetrating	General abdominal trauma	Blunt	22	Stable	Non-descriptive
							Penetrating	109		
Memon [18]	2013	Pakistan	Observational, Single center	2010–2012	Blunt	General abdominal trauma	None	32	Stable	Hemodynamic instability, severe internal bleeding, established peritonitis
Khubutiya [17]	2013	Russia	Observational, Single center	2000–2011	Blunt, penetrating	liver, spleen, stomach, small intestine, colon	Laparoscopy	155	stable	Hemodynamic instability, peritonitis, ongoing bleeding
							Laparotomy	106	unstable	
Huang [19]	2017	United States	Observational, Single center	2011–2014	Blunt	spleen	Laparoscopy	11	stable	<18 years old
							Laparotomy	41	stable	
Koto [23]	2018	South Africa	Observational, Single center	2012–2015	Blunt	Hollow viscus organ	Laparoscopy	27	stable	<12 years old
							Converted to laparotomy	8		
Parajuli [22]	2017	India	Observational, Single center	2008–2013	Blunt, penetrating	liver, spleen, stomach, small intestine, colon, mesentery, diaphragm	Blunt	48	stable	Hemodynamic instability, evisceration, gunshot wound
							Penetrating	71	stable	
Lin [20]	2018	Taiwan	Observational, Single center	2006–2015	Blunt	liver, spleen, stomach, small intestine, colon, mesentery, diaphragm, pancreas, duodenum, gall bladder, bladder	Laparoscopy	126	stable	Hemodynamic instability, FAST positive, attending surgeon’s decision
							Laparotomy	139	stable	
Matsevych [21]	2018	South Africa	Observational, Single center	2012–2015	Blunt, penetrating	General abdominal trauma	Blunt	8	stable	Non-descriptive
							Penetrating	33	stable	
Nicolau [24]	2019	Romania	Observational, Single center	2006–2016	Blunt, penetrating	liver, spleen, small intestine, colon, mesentery, diaphragm	Blunt	30	stable	Hemodynamic instability, <GCS 12; decompensated heart, lung or liver disease; major hemorrhage, organ evisceration, multiple major injuries, scarred abdomen
							Penetrating	20	stable	

FAST, focused assessment with sonography for trauma; GCS, Glasgow coma scale.

**Table 2:** NOS for the risk of bias and quality assessment of NRSs.

Author	Year	Selection				Comparability	Exposure			Total Score
		Adequate Definition of Patient Cases	Representativeness of Patient Cases	Selection of Controls	Definition of Controls	Control for Important or Additional Factors	Ascertainment of Exposure	Same method of Ascertainment for Participants	Nonresponse Rate	
Fabian [6]	1993	⋆	⋆	⋆			⋆	⋆		5
Townsend [7]	1993	⋆	⋆	⋆			⋆	⋆		5
Taner [8]	2001	⋆	⋆	⋆	⋆	⋆	⋆	⋆		7
Mathonnet [9]	2003	⋆	⋆	⋆			⋆	⋆		5
Omori [10]	2003	⋆	⋆	⋆	⋆	⋆	⋆	⋆		7
Mitsuhide [11]	2005	⋆	⋆	⋆			⋆	⋆		5
Huscher [12]	2006	⋆	⋆	⋆			⋆	⋆		5
Kaban [13]	2008	⋆	⋆	⋆	⋆	⋆	⋆	⋆		7
Mallat [14]	2008	⋆	⋆	⋆	⋆		⋆	⋆		6
Shah [15]	2011	⋆	⋆	⋆			⋆	⋆		5
Johnson [16]	2013	⋆	⋆	⋆	⋆	⋆	⋆	⋆		7
Memon [18]	2013	⋆	⋆	⋆			⋆	⋆		5
Khubutiya [17]	2013	⋆	⋆	⋆	⋆		⋆	⋆		6
Huang [19]	2017	⋆	⋆	⋆	⋆	⋆	⋆	⋆		7
Koto [23]	2018	⋆	⋆	⋆			⋆	⋆		5
Parajuli [22]	2017	⋆	⋆	⋆	⋆	⋆	⋆	⋆		7
Lin [20]	2018	⋆	⋆	⋆	⋆	⋆	⋆	⋆		7
Matsevych [21]	2018	⋆	⋆	⋆	⋆	⋆	⋆	⋆		7
Nicolau [24]	2019	⋆	⋆	⋆	⋆	⋆	⋆	⋆		7

NOS, Newcastle-Ottawa scale; NRS, non-randomized study; ⋆, The study has met the criteria for a domain of the Newcastle-Ottawa Scale.

**Table 3:** Subgroup analysis according to the prevalence of conversion to laparotomy, morbidity, and mortality.

Variable	Moderator	Number of Studies (*k*)	Proportion	95% CI	I^2^	Test for Subgroup Differences (Random Effect Model)	
Conversion to Laparotomy	Publication Year of Study					Q	*p*-Value
	before 2010	9	0.391	0.246; 0.556	37.7%	12.36	<0.001
	2011–present	6	0.115	0.067; 0.190	59.2%		
	Injured Organ						
	General abdominal organ	9	0.202	0.105; 0.353	82.3%	0.54	0.765
	Solid organ	2	0.265	0.073; 0.622	70.9%		
	Hollow viscus organ	4	0.319	0.085; 0.703	72.3%		
Morbidity	Publication year of study						
	before 2010	4	0.173	0.085; 0.318	27.5%	2.10	0.147
	2011–present	5	0.092	0.056; 0.149	0.0%		
	Injured Organ						
	General abdominal organ	6	0.092	0.044; 0.181	51.5%	2.43	0.297
	Solid	3	0.135	0.057; 0.286	0.0%		
	Hollow viscus organ	1	0.200	0.099; 0.364			
Mortality	Publication year of study						
	before 2010	6	0.000	0.000; 1.000	0.0%	0.00	0.999
	2011–present	6	0.019	0.004; 0.093	18.8%		
	Injured Organ						
	General abdominal organ	6	0.009	0.001; 0.067	0.0%	1.25	0.536
	Solid organ	3	0.000	0.000; 1.000	0.0%		
	Hollow viscus organ	3	0.053	0.005; 0.383	0.0%		

CI, confidence interval.

## Data Availability

Not applicable.
